# Burnout, Engagement, and Organizational Culture: Differences between Physicians and Nurses

**DOI:** 10.3889/oamjms.2015.091

**Published:** 2015-08-12

**Authors:** Dragan Mijakoski, Jovanka Karadzinska-Bislimovska, Vera Basarovska, Anthony Montgomery, Efharis Panagopoulou, Sasho Stoleski, Jordan Minov

**Affiliations:** 1 *Institute of Occupational Health of RM, WHO Collaborating Center, II Makedonska brigada 43, 1000 Skopje, Republic of Macedonia*; 2 *University of Macedonia, Thessaloniki, Greece*; 3 *School of Health Sciences, Department of Medicine, Aristotle University Thessaloniki, Thessaloniki, Greece*

**Keywords:** Burnout, Job engagement, Organizational culture, Work demands, Physician, Nurse

## Abstract

**BACKGROUND:**

Burnout results from a prolonged response to chronic emotional and interpersonal workplace stressors. The focus of research has been widened to job engagement.

**AIM:**

Purpose of the study was to examine associations between burnout, job engagement, work demands, and organisational culture (OC) and to demonstrate differences between physicians and nurses working in general hospital in Skopje, Republic of Macedonia.

**MATERIAL AND METHODS:**

Maslach Burnout Inventory and Utrecht Work Engagement Scale were used for assessment of burnout and job engagement. Work demands and OC were measured with Hospital Experience Scale and Competing Values Framework, respectively.

**RESULTS:**

Higher scores of dedication, hierarchy OC, and organizational work demands were found in physicians. Nurses demonstrated higher scores of clan OC. Burnout negatively correlated with clan and market OC in physicians and nurses. Job engagement positively correlated with clan and market OC in nurses. Different work demands were related to different dimensions of burnout and/or job engagement. Our findings support job demands-resources (JD-R) model (Demerouti and Bakker).

**CONCLUSIONS:**

Data obtained can be used in implementation of specific organizational interventions in the hospital setting. Providing adequate JD-R interaction can lead to prevention of burnout in health professionals (HPs) and contribute positively to better job engagement in HPs and higher quality of patient care.

## Introduction

Burnout is a psychological syndrome that results from a prolonged response to chronic emotional and interpersonal workplace stressors [1, 2].

In recent years, the focus of research on workplace psychosocial factors has been widened to the conceptual opposite of burnout: job engagement, which is defined as a positive work-related state of mind and considered to be at opposite pole [3]. However, Schaufeli and Bakker view job engagement as a separate phenomenon that can function independently of burnout [4]. The mechanisms of burnout and job engagement can be best understood within the demands/resources model of stress of Demerouti and Bakker (JD-R model) [5]. According to the model, job demands include physical (e.g., responsibility for too many patients, very fast work), social, emotional, cognitive, and organizational aspects of the work that require prolonged mental or physical efforts in workers and are therefore associated with emotional, cognitive, behavioral, and physiological changes, such as exhaustion and depersonalization. According to the JD-R model [5], job resources are those physical, psychological, social or organizational aspects of the work that help in achieving work goals, reducing job demands, and stimulating personal growth and development and protect workers from disengagement. Therefore, the JD-R model explains the development of burnout via: 1. demanding aspects of work (high job demands) leading to exhaustions, and 2. lack of resources resulting in withdrawal behavior (depersonalization) and disengagement. Following on from this, the demands and resources that individuals experience are footed in the organizational culture of the workplace.

The JD-R model expands the assumptions of the demand-control model (DCM) which proposes that control over the execution of tasks (autonomy) may buffer the impact of job demands on job stress. Hence, the JD-R model states that different types of job demands and job resources, depending on the organizational context, may interact in predicting job strain. It also accounts for more variance in emotional exhaustion and job satisfaction than DCM or effort-reward imbalance (ERI) model. The JD-R model, unlike DCM and ERI, focuses on both negative and positive indicators of employee well being. Therefore, it can be applied to a wide range of occupations, and can be used to improve employee well being and performance [6].

Organizational culture is a summary of the shared values, beliefs, or perceptions held by employees within an organization or organizational unit and it can influence the attitudes and behavior of the staff [7, 8]. This concept of organizational culture is particularly applicable in health care institutions, especially in hospital settings where performance depends on HPs’ interpersonal relationships. In this study we used the Competing Values Framework (CVF) [9] which is the most commonly used instrument for the assessment of organizational culture in health services research. CVF is based on the model that combines two dimensions “internal focus and integration versus external focus and differentiation” and “stability and control versus flexibility and discretion” and defines four archetypal cultures (clan, market, hierarchy, and adhocracy).

Clan culture exists in organizations that are perceived as pleasant places to work, where people like a family share a lot of personal information, and managers are seen as mentors. The organization that is result-oriented with competitive and goal-oriented employees is perceived as being market orientated.

Hierarchical culture is found in organizations that are perceived as very formalized and structured places to work with strict procedures, formal rules and policies, and managers are defined as good coordinators and organizers driven by efficiency of the organization.

The organization that is dynamic, entrepreneurial and creative place to work, where employees take risks and leaders are found to be innovators and risk takers is perceived as adhocracy.

In a French study conducted in 26 intensive care units significant relationships between organizational culture, burnout, and organizational performance were identified [10]. Results based on data from empirical studies using the CVF framework indicate that clan, adhocracy, and market cultures are differentially and positively associated with the effectiveness indices [11]. Moreover, research indicates that organizational culture can impact on quality of care and burned-out physicians demonstrate suboptimal patient care [12-14].

In the Republic of Macedonia the health care system faces continuous reforms aimed at the improvement of safety, effectiveness, patient-centeredness, timeliness, efficiency, and equity of patient care [15, 16]. However, these reforms and the social reforms (driven by EU accession targets) have raised complex hospital reorganisation issues that have resulted in increased demands in hospitals, for both physicians and nurses.

The purpose of the present study was to examine the associations between burnout, job engagement, work demands, and organizational culture and to examine differences between physicians and nurses working in the same Hospital.

## Methods

### Procedure

A cross-sectional study was conducted from November to December 2011 in a general Hospital in Skopje, R. Macedonia. The Hospital is a teaching hospital and an established educational base of the Medical Faculty of the University. Prior to the research, ethical approval was granted by the hospital management ethics committee. A contact person from the participating hospital was designated.

The hard copies of the questionnaires together with letter explaining the objectives of the study and assuring participants’ anonymity and confidentiality were distributed in envelopes to all physicians and nurses from the Hospital. Questionnaires were returned anonymously in sealed envelopes to protect participants’ privacy. Participation in the study was voluntary.

Prior to the actual study, within the pilot phase of FP7 ORCAB Project (http://orcab.web.auth.gr/), the questionnaires were validated among the sample of hospital health professionals from Greece, Turkey, Portugal, Romania, Bulgaria, Croatia, and Macedonia. The questionnaires were administered in-person and participants were asked to give their feedback on the face validity of the questionnaires, indicating if any of the questions were difficult to read, ambiguous or irrelevant to their work.

### Instruments

The survey tool consisted of standardised (Maslach Burnout Inventory, Utrecht Work Engagement Scale, and Competing Values Framework) and constructed-for-purpose questionnaires (Hospital Experience Scale).

Burnout was assessed using the Maslach Burnout Inventory (MBI) [17]. In this study emotional exhaustion (nine items) and depersonalisation (five items) subscales were used, and measured with a 7-point Likert scale (0 = never to 6 every day). Emotional exhaustion refers to feelings of overwhelming exhaustion and depletion of emotional resources. Depersonalization refers to the feelings of frustration, anger, and cynicism. It represents interpersonal dimension of burnout and it is described as an excessively detached response to other people. Emotional exhaustion was assessed through questions such as “I feel emotionally drained from my work” and “I feel burned out by my work”, and depersonalization with questions such as “I feel I treat some patients as if they were impersonal objects”. Responses are added to form a score for each subscale, thus giving each participant scores for the two components of burnout. The higher the score in one dimension means the higher level of burnout. The participants with the score of emotional exhaustion equal to 27 or higher or the score of depersonalization equal to 13 or higher were labelled as burnt out.

Job engagement was assessed with the Utrecht Work Engagement Scale (UWES) [18]. Vigor (six items) and dedication (five items) subscales were used, and were measured with a 7-point Likert scale (0 = never to 6 every day). High levels of mental energy and willingness to invest in work define vigour, whereas dedication refers to feelings of enthusiasm, pride and inspiration. Vigour was analysed with questions such as “At my work, I feel bursting with energy” and “I can continue working for very long periods at a time”, and dedication with questions such as “I find the work that I do full of meaning and purpose” and “My job inspires me”. Similarly to MBI, responses are added to form a score for each subscale. The higher the score in one dimension means the higher level of job engagement. The cut off points for high level of each job engagement subscale are: equal to 24 or higher for vigour and equal to 20 or higher for dedication.

Work demands, requiring prolonged mental or physical efforts in workers, were measured with the Hospital Experience Scale (HES), a questionnaire constructed for the purposes of FP7 ORCAB Project (http://orcab.web.auth.gr/) and the development of the HES was based on a qualitative analysis of focus groups (FGs). The purpose of FGs sessions, conducted in each ORCAB country (one for doctors, one for nurses, and one for residents and interns) was to understand what kind of stressors HPs are faced with. The FGs, made up of 6-8 participants, were audio recorded with the permission of the participants and later transcribed. The FGs texts were analysed, sample questions that related to main sources of stress were generated and they were used to develop HES items. The items were categorised according to the types of work demands: workload (seven items; ex., I am responsible for too many patients in hospital rounds), organisational (six items; ex., the roles in my department are not clear/ambiguous), emotional (six items; ex., I have to deal with verbally abusive patients) and cognitive (five items; ex., I have to take decisions when I don’t have all the information I need). Once these items were generated, the items were rated for relevance and appropriateness by a sample of HPs working in a general hospital. Extensive information on the validation of the HES can be provided upon request. Participants were asked to indicated their level of agreement with the items (1 = never to 5 = always). The mean score was calculated for the four types of work demands.

Organisational culture was measured with the Competing Values Framework (CVF) [19]. CVF is the most widely used and validated questionnaire for the assessment of organisational culture. The items of the CVF instrument (20 items) are divided equally into four subscales, each representing one of the four archetypal cultures (clan, market, hierarchy, and adhocracy). Therefore, each culture type is assessed by five items and each item defines certain attribute (dominant characteristics, leadership style, organization glue, strategic orientation, and system of rewards) of that culture type. As an example, items for the clan culture scale included: 1. the hospital is a very personal place; it is a lot like an extended family; people seem to share a lot of themselves; 2. managers in the hospital are warm and caring; they seek to develop employees’ full potential and act as their mentors or guides; 3. the glue that holds the hospital together is loyalty and tradition; commitment to this hospital is high; 4. the hospital emphasizes human resources; high cohesion and morale in the organization are important; and 5. the hospital distributes its rewards fairly equally among its members; it is important that everyone from top to bottom be treated as equally as possible. Participants were asked to indicated their level of agreement with the items (1 = strongly disagree, 2 = disagree, 3 = neither agree nor disagree, 4 = agree, 5 = strongly agree). The CVF can be scored in terms of strength of culture (number of points given to the attributes) and congruence (number of high scores given to the same culture type in relation to each attribute). In this study, points for statements relating to each of the four cultural types were averaged to derive the four culture types. There are no specific cut off points for this questionnaire and it was determined which of the four culture types got the highest mean value in different HPs’ profiles.

### Analysis

Independent samples *t* test and chi square test were used to compare groups (physicians and nurses).

During the data analysis we divided the total sample into two datasets - first including participants from medical specialties (Dermatology, Infectious Diseases, Intensive Care, Internal Medicine, Neurology, Physical Medicine and Rehabilitation, Radiology) and other including examinees from surgical specialties (Ophthalmology, ENT, Emergency Department, Surgery). Within these two datasets we also analysed differences between physicians and nurses.

Pearson’s correlation coefficients were calculated to examine relationships between continuous variables (burnout dimensions, job engagement components, different types of work demands, and organizational culture types). The Statistical Package for the Social Sciences (SPSS) statistics (Chicago USA 2011, version 20) was used for the statistical analyses.

## Results

Completed surveys were returned by 286 participants (138 physicians and 148 nurses). Physicians were 71 (51.4%) specialists, 38 (27.5%) interns, and 29 (21%) residents, while nurses were 116 (78.4%) senior nurses and 32 (21.6%) trainees. The response rate was 90.8% for physicians and 82.2% for nurses.

Physicians and nurses were not significantly different according to age while nurses had significantly longer tenure. Nurses also reported longer working hours per week than physicians (44.65 *vs*. 39.37, *p* < 0.001). The unusual data showing physicians working fewer hours than nurses prompted us to conduct additional interviews with the HPs from the Hospital. The majority of interviewed physicians stated that on the question about working hours per week they had put only working hours spent on direct patient care, not taking into consideration the hours spent on research, education, and consultations (additional 2-3 working hours per day).

Both genders were equally represented among physicians (men 52% *vs*. women 49%), but not for nurses (women 93%). The largest proportion of HPs in the sample worked in the Internal Medicine Department (physicians 27%, nurses 30%) and Surgery Department (physicians 35%, nurses 23%). Significantly more nurses were married (70.3% *vs*. 55.9%) (χ^2^(1) = 6.32, *p* = 0.012). Significantly more nurses (98.6%) than physicians (70.3%) reported full-time contract as a type of employment (χ^2^(1) = 44.96, *p* < 0.001).

Cronbach’s Alpha for cognitive demands in physicians, and emotional demands and cognitive demands in nurses were below 0.7, therefore they were deleted from further analyses.

Table 1 demonstrates that physicians showed significantly higher mean values on dedication (26.24 *vs*. 24.44, *t* = 2.34, *p* = 0.020), hierarchy type of organizational culture (3.27 *vs*. 2.86, *t* = 3.5, *p* = 0.001), and organizational work demands (2.51 *vs*. 2.24, *t* = 3.02, *p* = 0.003). On the other hand, nurses demonstrated significantly higher mean values for clan type of organizational culture (3.69 *vs*. 3.33, *t* = -3.2, *p* = 0.002). Despite the differences in organizational culture types between the two groups, the culture with the highest mean score for both physicians and nurses was clan type.

**Table 1:** Descriptive statistics of burnout, job engagement, organizational culture, and work demands and differences between physicians and nurses

		Mean	SD	Mean diff. (95% CI)	*p*
Burnout - Emotional Exhaustion	Physicians	15.28	11.56	-1.18 (-3.93, 1.57)	0.397

	Nurses	16.47	12.02		

Burnout - Depersonalization	Physicians	3.17	4.61	0.45 (-0.60, 1.50)	0.397

	Nurses	2.72	4.38		

Engagement – Vigor	Physicians	30.11	6.53	1.13 (-0.55, 2.81)	0.188

	Nurses	28.98	7.82		

Engagement – Dedication	Physicians	26.24	5.76	1.80 (0.29, 3.32)	0.020

	Nurses	24.44	7.19		

Organizational culture - Clan type	Physicians	3.33	0.94	-0.36 (-0.58, -0.14)	0.002

	Nurses	3.69	0.97		

Organizational culture - Market type	Physicians	3.16	0.89	0.04 (-0.19, 0.26)	0.750

	Nurses	3.13	1.01		

Organizational culture - Hierarchy type	Physicians	3.27	0.88	0.41 (0.18, 0.65)	0.001

	Nurses	2.86	1.12		

Organizational culture - Adhocracy type	Physicians	3.05	0.97	0.22 (-0.02, 0.47)	0.077

	Nurses	2.83	1.17		

Work demands - Physical demands	Physicians	3.25	0.68	-0.13 (-0.29, 0.02)	0.091

	Nurses	3.39	0.66		

Work demands - Organizational demands	Physicians	2.51	0.82	0.27 (0.10, 0.45)	0.003

	Nurses	2.24	0.71		

There were no significant differences between examined physicians and nurses with regard to high scores on the following variables: emotional exhaustion (equal to 27 or higher), depersonalization (equal to 13 or higher), vigour (equal to 24 or higher), and dedication (equal to 20 or higher).

Within the group of physicians we found no significant gender differences with regard to burnout, job engagement, work demands, and organizational culture. Given that 90% of examined nurses were women, no differences were analyzed.

Within two datasets (examinees from medical and surgical specialties) we found the same differences between physicians and nurses as differences registered within the total study sample.

Within the physicians group, participants who had a full-time contract showed significantly higher scores for clan type of organizational culture (3.49 *vs*. 2.95, *t* = 3.18, *p* = 0.002). Examinees with no full-time contract demonstrated significantly higher scores for organizational (3.16 *vs*. 2.23, *t* = -7.12, *p* < 0.001) and emotional (2.71 *vs*. 2.27, *t* = -4.0, *p* < 0.001) work demands as well as for hierarchy type of organizational culture (3.56 *vs*. 3.15, *t* = -2.95, *p* = 0.004).

Within the nurse group, only 1% of participants did not report having a full-time contract, thus no analyses were conducted.

Using bivariate analyses (Pearson’s correlation test), we found significant negative correlations of depersonalization with organizational culture (type clan) in physicians (see Table 2). On the other hand, emotional exhaustion in nurses was negatively correlated with organizational culture (type market) (see Table 3).

**Table 2:** Correlations of analyzed variables in examined physicians (*n* = 138)

	1	2	3	4	5	6	7	8	9	10	11
1. Emotional Exhaustion	0.897										

2. Depersonalization	0.628**	0.797								

3. Vigor	-0.332**	-0.359**	0.821								

4. Dedication	-0.287**	-0.319**	0.838**	0.908							

5. Organizational Culture – Clan	-0.125	-0.175*	0.132	0.092	0.821						

6. Organizational Culture – Market	-0.039	-0.146	0.051	0.087	0.566**	0.811					

7. Organizational Culture - Hierarchy	0.07	0.084	-0.028	-0.010	-0.061	0.253**	0.766				

8. Organizational Culture - Adhocracy	-0.069	-0.130	0.030	0.031	0.294**	0.616**	0.612**	0.838			

9. Work demands Physical demands	0.229**	0.088	-0.165	-0.148	-0.201*	-0.101	0.090	-0.047	0.740		

10. Work demands Organizational demands	0.104	0.126	-0.219*	-0.224**	-0.386**	-0.178*	0.111	-0.050	0.409**	0.798	

11. Work demands Emotional demands	0.248**	0.221**	-0.208*	-0.251**	-0.159	0.069	0.196*	0.126	0.378**	0.612**	0.694

** Correlation is significant at the 0.01 level (2-tailed)

* Correlation is significant at the 0.05 level (2-tailed)

*Note*. Cronbach’s alpha on the diagonal. First row numbers (1-11) indicate the same respective variables as shown in the first column (eg., 1. Emotional Exhaustion, 2. Depersonalization, 3. Vigor, etc.).

**Table 3:** Correlations of analyzed variables in study nurses (*n* = 148)

	1	2	3	4	5	6	7	8	9	10
1. Emotional Exhaustion	0.880									

2. Depersonalization	0.631**	0.717								

3. Vigor	-0.250**	-0.271**	0.825							

4. Dedication	-0.269**	-0.282**	0.827**	0.888						

5. Organizational Culture – Clan	-0.081	-0.097	0.065	0.180*	0.805					

6. Organizational Culture – Market	-0.217**	-0.001	0.134	0.222**	0.216**	0.804				

7. Organizational Culture – Hierarchy	-0.185*	-0.027	0.071	0.106	0.001	0.576**	0.840			

8. Organizational Culture – Adhocracy	-0.244**	-0.094	0.083	0.113	0.076	0.487**	0.760**	0.868		

9. Work demands Physical demands	0.336**	0.200*	-0.083	-0.090	-0.219**	-0.089	-0.072	-0.121	0.723	

10. Work demands Organizational demands	0.387**	0.272**	-0.226**	-0.316**	-0.382**	-0.24**	-0.176*	-0.30**	0.495**	0.750

** Correlation is significant at the 0.01 level (2-tailed)

* Correlation is significant at the 0.05 level (2-tailed)

*Note*. Cronbach’s alpha on the diagonal. First row numbers (1-10) indicate the same respective variables as shown in the first column (eg., 1. Emotional Exhaustion, 2. Depersonalization, 3. Vigor, etc.).

Dedication and total job engagement score in nurses were positively correlated with organizational culture (type market). There was also a significant positive correlation of dedication with organizational culture (type clan) in nurses. In both groups there was significant positive correlation between vigour, dedication, and total engagement score.

Physical work demands were positively correlated only with emotional exhaustion in physicians, whereas in nurses there were significant positive correlations with both emotional exhaustion and depersonalisation.

Within the physicians group, organizational work demands showed significant negative correlation with job engagement dimensions. In nurses, organizational work demands were significantly correlated with either burnout (positive correlation) or job engagement (negative correlation).

Emotional work demands were correlated with both burnout dimensions and job engagement scores in physicians.

## Discussion

Physicians reported higher scores for dedication and organizational work demands, while nurses reported higher scores for clan type of organizational culture. Such analyses regarding health professionals and their work context are rare for Macedonian hospitals. The high response rate means that we have an opportunity to ascertain a comprehensive picture of burnout and engagement in this organisation.

Significantly higher scores of dedication in physicians underline the potentially positive effects of work in this group of HPs and suggest the aspects of work that could reduce job demands and the associated physiological and psychological costs [3]. In accordance with JD-R Model, increased organizational work demands in physicians could increase compensatory efforts in order to maintain performance level (dedication and job engagement in total).

Organizational culture and safety climate are determined as important factors of healthcare worker well-being and patient safety [20].

In this study we found differences between physicians and nurses in their beliefs and perceptions about the characteristics or values of the organization. This study shows that contrary to nurses, physicians perceived hospital organizational culture as more bureaucratic. On the other hand, nurses perceived that hospital interpersonal relationships were characterised by cohesion, personnel was working as a team, and leadership was acting as a mentor who promoted human resources development. But, despite the differences between the two groups, the culture type with the highest mean score in both physicians and nurses was clan. Similarly, a cross-sectional study conducted in Netherlands in 83 health professionals involved in diabetes care showed clan as type of organizational culture with the highest mean score [21].

In this study there were no differences between physicians and nurses with regard to emotional exhaustion, depersonalization, vigour, and total job engagement.

Contrary to our study (about 18% of nurses reported high emotional exhaustion), US data has reported that 43% of the nurses had high emotional exhaustion scores and a similar proportion was dissatisfied with their current jobs [22]. Participants in the current study also demonstrated lower average emotional exhaustion (physicians - 15.3, nurses - 16.5) and depersonalisation (physicians - 3.2, nurses - 2.7) scores unlike nurses from 40 units in 20 urban hospitals across the United States (emotional exhaustion - 24.3, depersonalization - 7.4) [23].

Despite findings that physicians reported fewer working hours than nurses additional interviews with physicians indicated that their reported hours did not include additional working hours spent on research, education, and consultations. Therefore, differences in burnout scores between the actual study and other studies cited could not be attributed to shorter working hours in health professionals from this research. In fact, health care reforms in the country have increased work demands for both physicians and nurses from this hospital. However, lower average emotional exhaustion and depersonalisation scores in the actual research could be explained by teamwork, excellent interpersonal relationships, support from superiors and co-workers, independence in decision-making, and appropriate physical conditions previously detected as protective workplace factors (or job resources) through qualitative analysis of focus groups’ data [15]. Additionally, this study also showed that clan type was the organizational culture with the highest mean score in both physicians and nurses, having certain role in protecting workers from burnout.

No gender differences among physicians were found, contrary to similar studies in Turkey [24], and the US [25]. However, research indicates that gender is not a consistent predictor of burnout, with studies reporting mixed results [26-28].

This study employed the CVF instrument as the most commonly used tool for organizational culture assessment in health services research. There was a negative correlation between depersonalization with organizational culture (clan type) in physicians, while there was a negative correlation between emotional exhaustion with organizational culture (market type) among nurses.

Similarly, Linzer et al. in 2009 showed that adverse workflow (time pressure and chaotic environments), low work control, and unfavourable organizational culture were strongly associated with low physician satisfaction, high stress, burnout, and intent to leave [29]. Demir et al. also reported that problematic relationships among team members were shown to increase burnout [30].

This study demonstrated that physical work demands were positively correlated only with emotional exhaustion in physicians, whereas in nurses there was significant positive correlation with both emotional exhaustion and depersonalization. Within the physicians group, organizational work demands showed significant negative correlation with job engagement dimensions. In nurses, organizational work demands were significantly correlated with burnout (positive correlation) and job engagement (negative correlation).

Emotional work demands were correlated with both burnout dimensions and job engagement scores in physicians. Similarly to our findings, research has demonstrated that physicians who perceive greater control over the practice environment, who perceive that their work demands are reasonable, and who have more support from colleagues have higher levels of satisfaction, commitment, and psychological well-being [31]. In a survey among 212 health care workers at a hospital in Norway it was demonstrated that in addition to emotional exhaustion, both work demands and organizational supports had direct effects on work attitudes (job satisfaction and organizational commitment) [32].

Correlations between work demands and burnout and job engagement dimensions are consistent with the JD-R Model which explains that job demands are associated with emotional, cognitive, behavioural, and physiological changes in workers, such as exhaustion and depersonalization (or disengagement) [5].

We can conclude that this study shows clear associations between burnout, job engagement, organizational culture, and work demands, and differences between physicians and nurses working in the same hospital.

In contrast with the burnout literature, we found lower average emotional exhaustion and depersonalisation scores as well as low frequency of participants with high emotional exhaustion. Probably the main role for such circumstances is hospital “protective factors” (e.g., teamwork, excellent interpersonal relationships, support from superiors and co-workers, independence in decision-making, and appropriate physical conditions).

The data obtained can be used in the creation and implementation of specific organizational interventions in the hospital setting, guided by the differences found in work demands and different perceptions of organizational culture in physicians and nurses. Providing adequate job demands-resources interaction can lead to the prevention of work-related burnout in HPs, and contribute positively to better job engagement in HPs and higher quality of patient care.

Findings of this study should be interpreted with caution as cross-sectional research is limited with regard to causality. Also, a “healthy worker effect” may mean that we have under-estimated the levels of burnout in physicians and nurses. Despite the small sample size, the high response rate gave us an opportunity to ascertain a comprehensive picture of burnout and engagement in both physicians and nurses working in a same Hospital.

## Funding

This work was supported by the European Union’s Seventh Framework Programme [FP7-HEALTH-2009-single-stage] [grant agreement no. 242084].
